# FoxP3^−^ Tr1 Cell in Generalized Myasthenia Gravis and Its Relationship With the Anti-AChR Antibody and Immunomodulatory Cytokines

**DOI:** 10.3389/fneur.2021.755356

**Published:** 2021-12-17

**Authors:** Huanyu Meng, Shuyu Zheng, Qinming Zhou, Yining Gao, You Ni, Huafeng Liang, Sheng Chen

**Affiliations:** ^1^Department of Neurology, Ruijin Hospital, Shanghai Jiaotong University School of Medicine, Shanghai, China; ^2^Brain Injury Center, Renji Hospital, Shanghai Jiaotong University School of Medicine, Shanghai, China; ^3^Department of Neurology, Xinrui Hospital, Wuxi, China

**Keywords:** Tr1 cells, myasthenia gravis, AChR ab, Foxp3, interleukin 10

## Abstract

**Introduction:** The changes in the number and function of regulatory T cells (Tregs) are thought to play important roles in the pathogenesis of generalized myasthenia gravis (gMG). Previous studies have suggested the decrease of FoxP3^+^ Treg cells in the MG development. However, there is no study on the pathophysiological mechanism of FoxP3^−^Treg, especially Tr1 cells, in gMG patients. Therefore, this study was conducted to reveal the effect of Tr1 cells to the pathophysiology of gMG.

**Methods:** Thirteen patients with gMG and twelve healthy volunteers were enrolled in this study. The titer of anti-AChR Ab was measured by ELISA. The separated PBMCs were labeled for CD4, CD25, CD49b, LAG3 and FoxP3. The CD4^+^ T cell count, FoxP3^+^ Treg to CD4^+^ T cell ratio and Tr1 cell to CD4^+^ T cell ratio were measured by flow cytometry. Based on the FoxP3^+^ Treg and Tr1 cell to CD4^+^ T cell ratios, the patients' Tr1 cell to FoxP3^+^ Treg ratios were calculated. The IL-6, IL-7, IL-10, TGF-β and IFN-γ concentration in the serum of MG patients and normal controls (NCs) were measured via ELISA.

**Results:** We found a significantly positive correlation between the Tr1 cell/CD4^+^ T cell ratio and the anti-AChR Ab (*r* = 0.6889 ± 0.4414, *p* = 0.0401). Although there were no significant differences in the relationship between FoxP3^+^ Treg cells and anti-AChR Ab, a positive correlation between the Tr1 cell/FoxP3^+^ Treg cell ratio and the anti-AChR Ab (*r* = 0.7110 ± 0.4227, *p* = 0.0318) was observed. In addition, the Tr1 cell/CD4^+^ T cell ratio but not the proportion of FoxP3^+^ Tregs was positively correlated with IL-10 (*p* = 0.048). These results suggested that in the process of the immunomodulatory effect of Tr1 cells in patients with gMG, IL-10 and other cytokines may be involved, but the specific mechanism needs further study.

**Conclusion:** This is the first study of the immunoregulatory mechanism of Tr1 cells in gMG. We conducted this study to elucidate the significance of Tr1 cells in the pathogenesis of MG. We believe that in patients with gMG, Tr1 cells may play an immunomodulatory role in counteracting AChR-related autoimmune responses. In this process, IL-10 and other immunomodulatory cytokines may be involved.

## Introduction

Myasthenia gravis (MG) is an autoimmune disease characterized by fatigue, ptosis, dysphagia and dyspnea, which are mediated by multiple antibodies blocking nerve impulse transmission at the neuromuscular junction (1, 2). Although the pathogenesis of MG is complex and the factors initiating autoimmunity are largely unknown, previous studies have suggested that the decline in immune regulatory cell function and concentration is important in the pathogenesis of MG (3). As a principal player in peripheral tolerance, regulatory T (Tregs) are among the most widely studied of regulatory cells, mainly including CD4^+^CD25^+^FoxP3^+^ Tregs (4), γδT cells (5), CD8^+^ T cells, CD4^+^CD25^−^FoxP3^−^CD49b^+^LAG3^+^ type 1 regulatory T (Tr1) cells (6, 7) and so on. Among these cells, FoxP3^+^ Tregs are well-known for their immunosuppressive functions. To be specific, Tregs can suppress potential autoreactive T cells and protect the body from CD4^+^ T cell-mediated autoimmune diseases (2, 3). The dysfunction or decrease in the number of FoxP3^+^ Tregs is believed to play a pivotal role in the pathogenesis of MG by perturbing T cell tolerance (4, 8). However, some previous studies exploring the percentages of FoxP3^+^ Tregs within CD4^+^ T cells have not shown significant differences between MG patients and healthy controls, generating controversial results (2, 9).

In addition to FoxP3^+^ Treg cells, there are other types of CD4^+^ Treg cells named FoxP3^−^ Tr1 cells. By defining subpopulations of Tregs, some studies detected a significant increase in the frequency of CD45RA^+^FoxP3 low-expressing cells in MG patients (3). However, the role of Tr1 cells in the pathogenesis of MG has not been reported. Tr1 cells are induced in the periphery and play a pivotal role in promoting and maintaining T cell tolerance (4). It is known that the level of Tr1 may vary with immune microenvironment, leading to changes in the types and levels of cytokines secreted (10).

Furthermore, elevated levels of proinflammatory cytokines, such as IL-6, IL-17, and IFN-γ, secreted by T effector cells (Teff) have been observed in MG patients (1). Via contact-dependent and contact-independent suppression of Teff cells, Tregs normally suppress the production of these proinflammatory cytokines (1, 11). After activation, similar to FoxP3^+^ Tregs, a proportion of Tr1 cells can also produce cytokines related to immune regulation, including TGF-β and IL-10, and participate in immune regulation in patients with MG (11). However, whether FoxP3^+^ Tregs or Tr1 cells play a major role in the changes in the levels of immune regulatory cytokines in patients with MG has not yet been determined.

Therefore, this study analyzed the FoxP3^+^ Treg and Tr1 concentrations, as well as the levels of anti-AChR Ab and anti-inflammatory cytokines, in the peripheral blood of patients with MG and healthy controls. This study was conducted to elucidate the significance of Tr1 cells in the pathogenesis of MG and to provide a theoretical basis for subsequent possible immunosuppressive treatment.

## Materials and Methods

### Patients

Thirteen anti-AChR Ab-positive MG patients from the Department of Neurology of Ruijin Hospital since 2019 and twelve healthy volunteers were enrolled in this study. All the patients with MG included in the study were patients with new-onset MG, without a history of immunosuppressant use, or previous history of autoimmune diseases. Healthy volunteers needed to meet the requirements of no acute infectious diseases, no previous history of autoimmune diseases, and no history of immunosuppressant usage. The medication histories of all the myasthenia gravis patients enrolled in this study were recorded. The patients' peripheral blood samples were collected to evaluate the levels of anti-AChR Ab in the patients' sera. Autoimmune antibodies, including anti-AChR Ab were measured by ELISA. This study was approved by the Ethics Review Committees of Ruijin Hospital, Shanghai Jiao Tong University School of Medicine. All participants provided informed consent.

### Flow Cytometry

**Figure 1:**
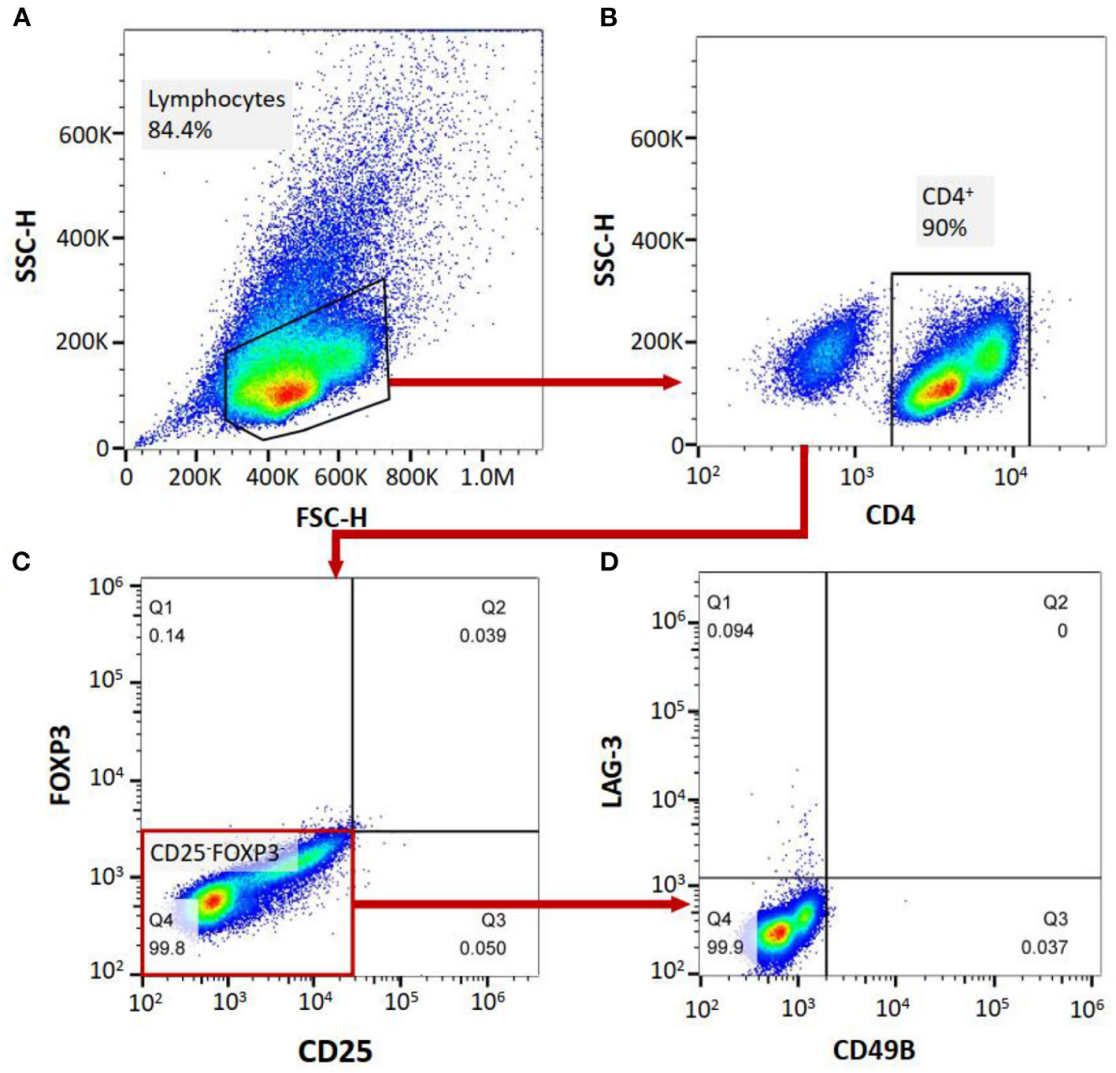
**(A–D)**, labeling PBMCs with anti-CD4, anti-CD25, anti-FoxP3, anti-CD49B and anti-LAG-3 antibodies, and cell subgroup identification with flow cytometry.

Flow cytometry was performed on a FACS-Canto II system (BD Biosciences, San Jose, CA, USA). Ten milliliters of peripheral blood were collected from MG patients and healthy controls to complete the flow cytometric analysis of the cell subgroups in the peripheral blood of the patients. The PBMCs were separated from the peripheral blood of the patients. The peripheral blood mononuclear cells (PBMCs) or isolated Tregs were analyzed for the expression of surface or intracellular markers using the following anti-human antibodies: PerCP mouse anti-human CD4 (clone: L200, BD Biosciences), PE-Cy™7 mouse anti-human CD25 (clone: M-A251, BD Biosciences), FITC mouse anti-human CD49b (clone: AK-7, BD Biosciences), Alexa Fluor® 647 mouse anti-human LAG-3 (BD Biosciences), and PE mouse anti-human FoxP3 (clone: 259D/C7, BD Biosciences). For surface marker staining, the cells were incubated with the antibodies for 30 min at 4°C in the dark and then washed with PBS. After staining, the cells were washed and fixed with 1% paraformaldehyde. The samples were divided into two aliquots, one treated with corresponding antibodies and the other with isotype antibodies as negative control. For gating strategy, cells were firstly gated on FSC/SCC to choose lymphocyte population, subsequently analyzing for CD4 expression. CD4+ cells were selected and then assessed for the expression of CD25 and Foxp3 on a quadrant. We then analyzed CD49b and LAG3 expression on CD25-Foxp3- cell population to determine CD4^+^CD25^−^FoxP3^−^CD49b^+^LAG3^+^ T cells (Tr1 cells). All the data were analyzed by FlowJo software VX. The CD4^+^ T cell count, FoxP3^+^ Treg/CD4^+^ T cell ratio and Tr1 cell/CD4^+^ T cell ratio were measured. Based on the FoxP3^+^ Tregs/CD4^+^ T cell ratio and Tr1 cell/CD4^+^ T cell ratio, the Tr1 cell/FoxP3^+^ Treg ratio of the patients and NCs was calculated (Figure 1).

### ELISA

The IL-6, IL-7, IL-10, IFN-γ, TGF-β and anti-AchR antibodies levels in the sera of the MG patients and NCs were measured via ELISA Kits (EK0410, EK0779, EK0416, EK0373, EK0513 and ElisaRSR^TM^ AChRAb, respectively, from Boster Biological Technology, Pleasanton, CA, USA), in accordance with manufacturer instructions. In particular, the cut-off value for anti-AchR antibody positive was 0.45 nmol/L.

### Statistical Analysis

**Figure 2:**
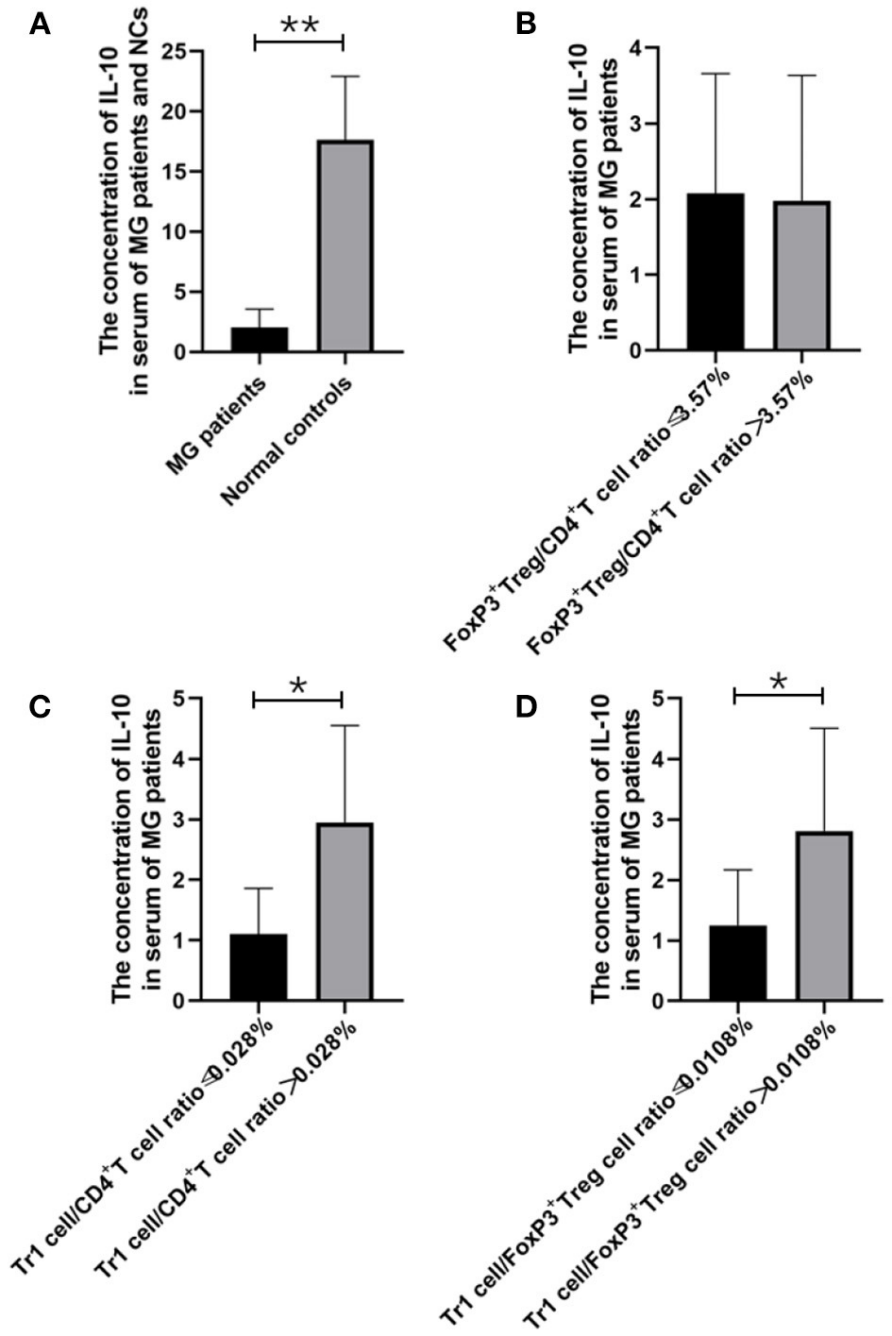
The relationship between IL-10 concentration and MG onsets, FoxP3^+^ Treg/CD4^+^T ratio, Tr1/CD4^+^T cells ratio and Tr1/FoxP3^+^ Tregs ratio. **(A)**, IL-10 concentration in the serum of MG patients was found significantly lower than the concentration of IL-10 in the NCs (*p* = 0.005). **(B–D)**, higher Tr1/CD4^+^T cells ratio and Tr1/FoxP3^+^ Tregs ratio were usually accompanied by higher IL-10 concentration, and a significant positive correlation was found between Tr1/ CD4^+^T ratio and IL-10 (*p* = 0.048), Furthermore, Tr1/FoxP3^+^ Tregs cell ratio was found positively correlated with IL-10 (*p* = 0.048). The data were shown as mean with ± SD of each group, *means *p* < 0.05, **means *p* < 0.01. *N* = 10 for MG group and *N* = 8 for NC group.

**Figure 3:**
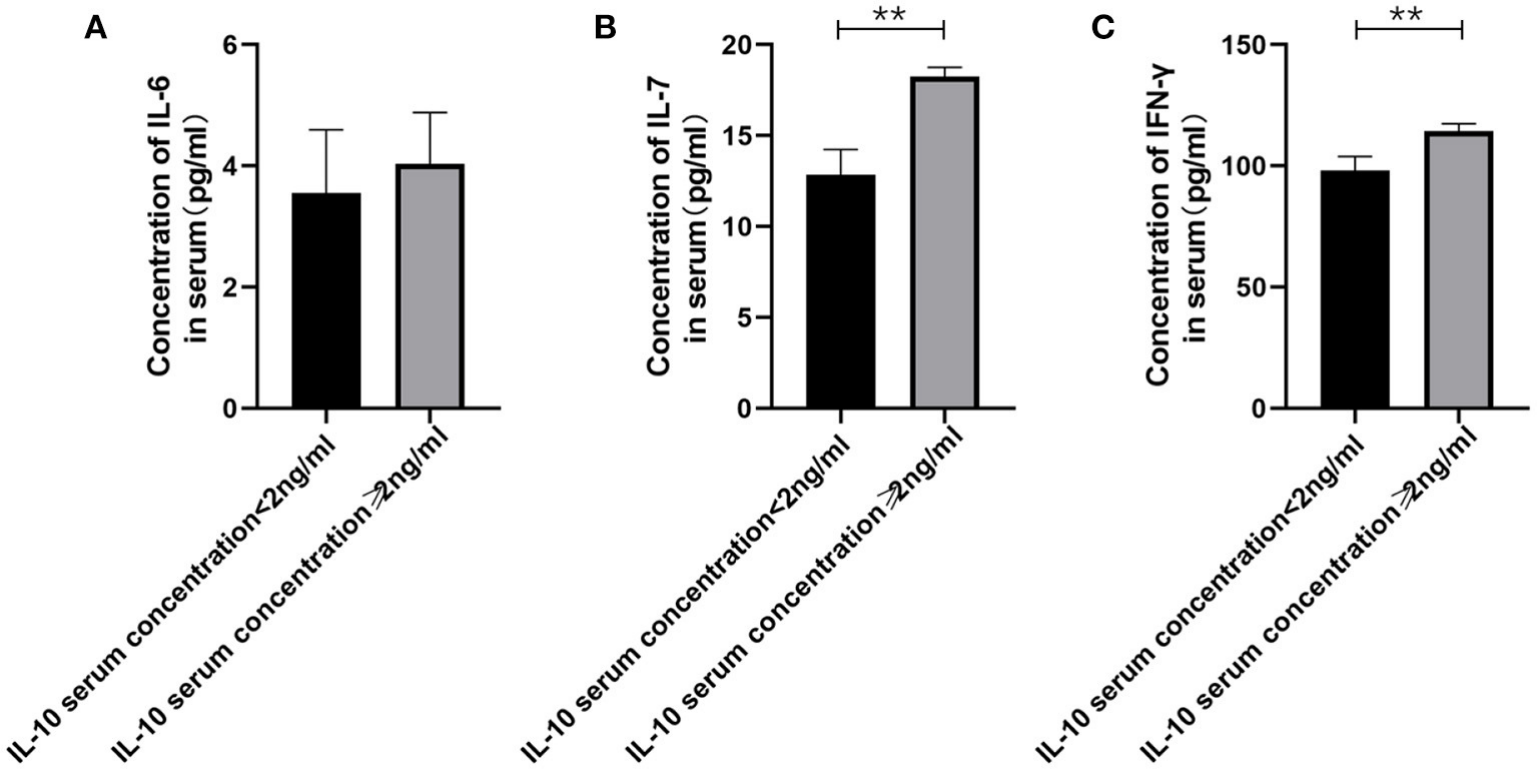
The relationship between IL-10 levels in patients with gMG and the levels of pro-inflammatory cytokines including IL-6, IL-7 and IFN-γ. **(A)**, patients with high serum IL-10 levels were usually accompanied by higher IL-6 concentration, but there was no significant difference in serum IL-6 levels between patients with high IL-10 levels and low IL-10 levels (*p* = 0.443). **(B)**, Generalized MG patients with low IL-10 levels were usually accompanied by a significant decrease in IL-7 levels (*p* < 0.001). **(C)**, Generalized MG patients with low IL-10 levels were usually accompanied by a significant decrease in IFN-γ levels (*p* = 0.001). The data were shown as mean with ± SD of each group, *means *p* < 0.05, **means *p* < 0.01. *N* = 5 for each group within gMG patients.

**Figure 4:**
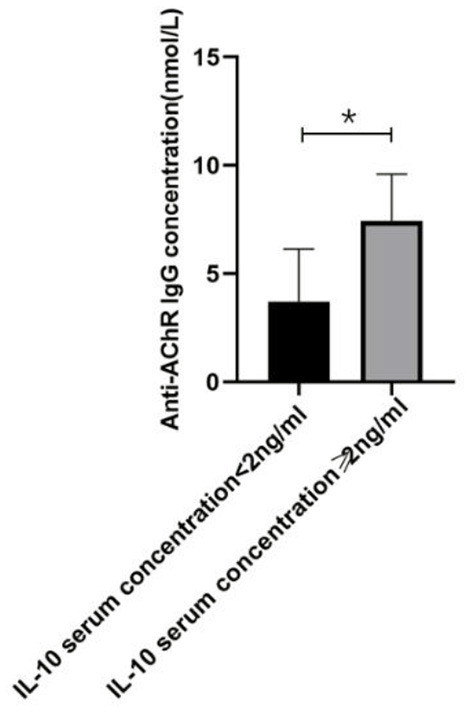
The relationship between IL-10 and anti-AChR Ab concentration. There was a significant difference in the anti-AChR Ab levels between gMG patient groups with low and high IL-10 concentrations. Generalized MG patients with high IL-10 concentration was usually accompanied by a significant increase in anti-AChR Ab levels (*p* = 0.034). The data were shown as mean with ± SD of each group, *means *p* < 0.05, **means *p* < 0.01. *N* = 5 for each group within gMG patients.

The clinical data analysis was performed by SPSS software (version 23.0 for Windows; SPSS Inc., IL, USA). Comparisons between different groups of MG patients and healthy controls were analyzed using the two-tailed unpaired Student's *t*-test and Chi square tests. The correlation was evaluated using Spearman's rho. A *p*-value < 0.05 was considered significant. The cut-off values for dividing two groups in Figures 2–4 were determined based on the median value for continuous variables. Because of the problem of the samples, several samples in MG/NC group were omitted. All the statistical analyses were performed using GraphPad Prism software (version 5.0).

## Results

### Differences in the FoxP3^+^ Tregs, Tr1 Cells, and Tr1 cell/FoxP3^+^ Treg Ratio in the PBMCs of MG Patients and NCs

Among the MG patients and normal controls (NCs) (*n* = 25), including 13 MG patients and 12 NCs, we found significant differences in anti-AChR Ab titer (*p* < 0.001), IL-7 (*p* < 0.001), IL-10 (*p* = 0.005) and IFN-γ (*p* = 0.007), but not in age, sex, FoxP3^+^ Treg/CD4^+^T ratio, Tr1 Treg/CD4^+^T ratio, Tr1/FoxP3^+^ Treg ratio, TGFβ1 and IL-6 titer, between MG patients and normal individuals. Among the enrolled MG patients, all patients were confirmed to be gMG with no history of immunosuppressants use. Three patients were eligible for myasthenia gravis crisis. The remaining 10 patients were patients with type 2B myasthenia gravis (Table 1).

**Table 1:** The basic clinical characteristics of the subjects enrolled in this study.

		MG patients (^*^)	NCs (^*^)	***p***	**Total**
Age (range, mean ± SD)		55.23 ± 18.34	47.58 ± 12.19	0.236	51.56 ± 15.86
Sex [*n*, (%)]	Male	6	5	0.830	11
	Female	7	7		14
Anti-AChR Ab (nmol/L, mean ± SD)		5.57 ± 2.92 (*n* = 13)	0.01 ± 0.03 (*n* = 11)	<0.001	2.94 ± 3.52
FoxP3^+^ Treg/CD4^+^T ratio (mean ± SD)		3.22% ± 2.70 (*n* = 10)	4.30% ± 2.92 (*n* = 11)	0.347	3.70 ± 2.78
Tr1 Treg/CD4^+^T ratio (mean ± SD)		0.02 ± 0.18% (*n* = 10)	0.10% ± 0.03 (*n* = 11)	0.217	0.07% ± 0.14
Tr1/FoxP3^+^ Treg ratio (mean ± SD)		0.01 ± 0.07% (*n* = 10)	0.02% ± 0.18 (*n* = 11)	0.589	0.06% ± 0.13
TGFβ1 (mean ± SD)		98.21 ± 138.33 (*n* = 10)	119.52 ± 179.33 (*n* = 8)	0.641	108.31 ± 154.35
IL-6 (mean ± SD)		3.79 ± 0.93 (*n* = 10)	15.44 ± 19.01 (*n* = 8)	0.277	8.97 ± 13.60
IL-7 (mean ± SD)		15.56 ± 3.00 (*n* = 10)	1.68 ± 3.74 (*n* = 8)	<0.001	8.98 ± 7.84
IL-10 (mean ± SD)		2.02 ± 1.53 (*n* = 10)	28.72 ± 26.52 (*n* = 8)	0.005	14.67 ± 22.39
IFN-γ (mean ± SD)		106.26 ± 9.53 (*n* = 10)	49.13 ± 58.47 (*n* = 8)	0.007	79.20 ± 49.23

* *Some measurements were missing due to the unavailability of the patients and loss to follow-up*.

Similar to previous research results, there was no significant difference in the FoxP3^+^ Treg/CD4^+^ T cell ratio (median values 3.22 vs. 4.30%, *p* = 0.347) between the MG patients and NCs enrolled in our study. As important immunoregulatory cells, Tr1 cells play an important role in the pathophysiology of MG (12). However, the Tr1/CD4^+^ T cell ratio and the Tr1/FoxP3^+^ Treg cell ratio in MG patients tend to decrease compared with normal controls, but there were also no significant differences in the proportion of Tr1 cells to CD4^+^ T cells (median values 0.02 vs. 0.10%, *p* = 0.217) or in the Tr1 cell/FoxP3^+^ Treg ratio (median values 0.01 vs. 0.02%, *p* = 0.589) between the MG patients and NCs.

### The Relationship Between Anti-AChR Ab Levels and FoxP3^+^ Tregs and Tr1 Cells

There was no significant difference in the FoxP3^+^ Treg/CD4^+^ T cell ratio (*p* = 0.347), Tr1 cell/CD4^+^ T cell ratio (*p* = 0.178) or Tr1 cell/FoxP3^+^ Treg ratio (*p* = 0.589) between the MG patients and NCs. To characterize the relationship among the FoxP3^+^ Treg/CD4^+^ T cell ratio, Tr1 cell/FoxP3^+^ Treg ratio, Tr1 cell/FoxP3^+^ Treg ratio and the anti-AChR Ab concentration, we further dividing the MG patients into 2 subgroups based on the median of anti-AChR Ab concentration as cut-off values. Interestingly, in the patients within MG subgroups included in this study, there was no significant relationship between the FoxP3^+^ Treg cells and anti-AChR Ab levels, but there was a positive correlation with statistically significant between the Tr1 cell/CD4^+^ T cell ratio and the anti-AChR Ab level (*r* = 0.6889 ± 0.4414, *p* = 0.0401). In addition, there was also a positive correlation between the Tr1 cell/FoxP3^+^ Treg cell ratio and the anti-AChR Ab level (*r* = 0.7110 ± 0.4227, *p* = 0.0318).

### The Relationship Between TGF-β and IL-10 and Different Treg Cell Subsets

We found that the Tr1 cell/CD4^+^ T cell ratio and Tr1 cell/FoxP3^+^ Treg cell ratio are significantly related to the titer of anti-AChR Ab, but how Tr1 cells mediate changes in the immune environment is still not very clear. In this study, IL-10 concentration in the serum of MG patients is found significantly lower than the concentration of IL-10 in the NCs (2.02 vs. 28.72 pg/ml, *p* = 0.005). There was no significant correlation between FoxP3^+^ Treg and the TGF-β (*p* = 0.416) and IL-10 (*p* = 0.928) levels in the patients with MG, but it is interesting that although there was no significant correlation between Tr1 cells and TGF-β concentrations (*p* = 0.371), Tr1 cells were positively correlated with IL-10 levels (*p* = 0.048).

Therefore, we have analyzed the levels of IL-10 and other immune-related cytokines. It is currently believed that IL-10 mainly acts on the feedback regulation stage of the immune pathway of patients with autoimmune diseases, and its serum levels are usually related to other proinflammatory cytokines (13, 14). We found that a similar situation exists in patients with gMG. The level of IL-10 is positively correlated with the levels of cytokines such as IL-7 (*p* < 0.001) and IFN-γ (*p* = 0.001), but not IL-6 (*p* = 0.443).

In the present study, we respectively described the relationship between Tr1 cell levels and anti- anti-AChR Ab levels in patients with gMG, and the relationship between Tr1 cells and IL-10 and other immunomodulatory cytokines. However, this could only indicate that in patients with gMG, Tr1 cell levels are positively correlated with anti-AChR Ab levels and IL-10 levels, respectively. We found that IL-10 concentration is also positively correlated with anti-AChR Ab levels (*p* = 0.034). But whether Tr1 cells could regulate anti-AChR Ab level through the secretion of high levels of IL-10 has not been proved, and further research is needed.

## Discussion

MG is an autoimmune disorder characterized by muscle weakness and chronic fatigue that results from a blockage of nerve impulse transmission by anti-AChR Ab at the neuromuscular junction (1). However, to date, the exact etiology and pathogenesis of MG remain unclear (2). Previous studies have shown that T cells may play an important role in pathogenesis of MG (3).

The two most studied regulatory T cell subtypes are FoxP3^+^ Tregs and Tr1 cells (11). As the most important regulatory T cells, FoxP3^+^ Tregs were firstly defined by Sakaguchi in 1995 and are characterized by expressing CD25 and FoxP3 (2). In addition to FoxP3^+^ Tregs, Tr1 cells are induced in the periphery and play a pivotal role in promoting and maintaining T cell tolerance (4). Regulatory T cell-mediated suppression serves as a pivotal mechanism of the negative regulation of immune-mediated inflammation (4). To be specific, FoxP3^+^ Tregs can suppress potential autoreactive T cells and protect the body from CD4^+^ T cell-mediated autoimmune diseases (2). As an important subset of CD4^+^ T cells, Tr1 cells could help control excessive inflammatory responses and maintain tolerance (15). Tr1 cells prevent and downregulate undesirable immune responses to pathogenic and non-pathogenic antigens and are associated with long-term tolerance in many human conditions (10, 16, 17).

**Figure 5:**
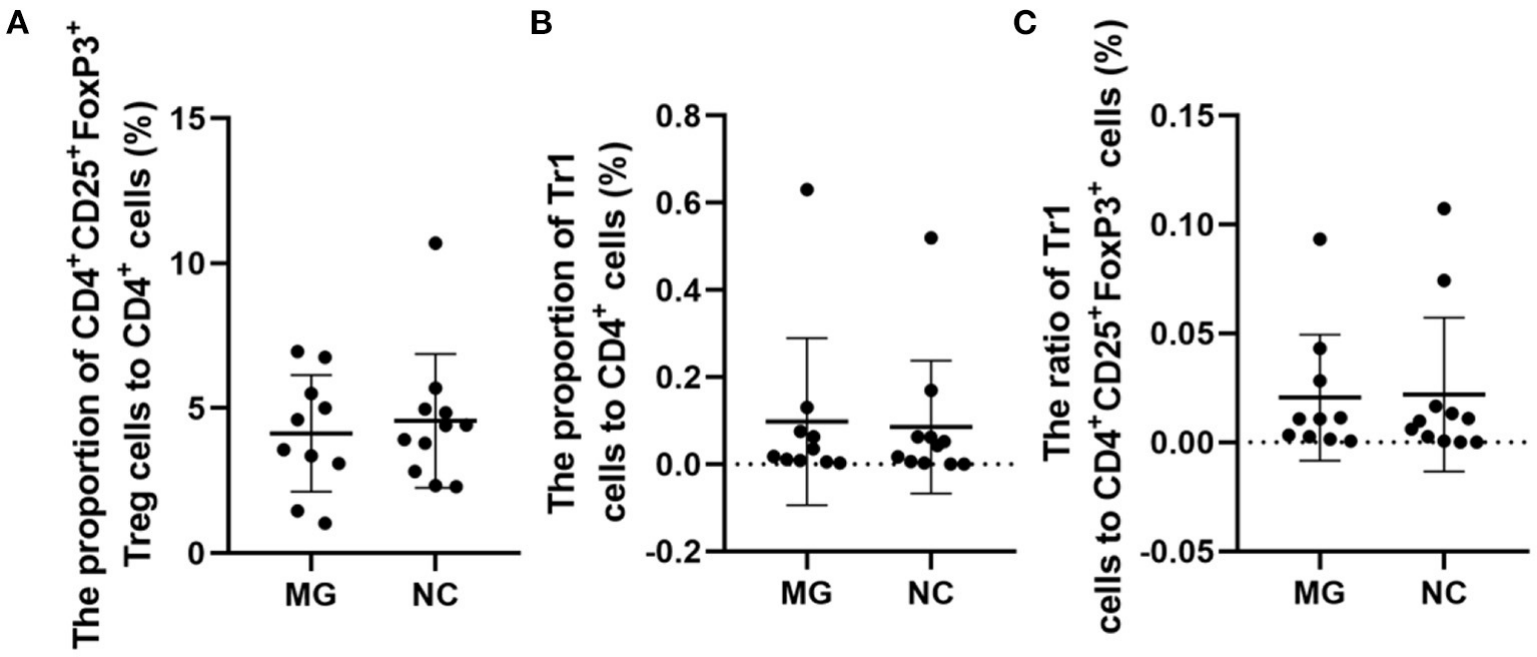
Frequencies of FoxP3^+^ Tregs in MG patients compared to NCs. **(A–C)**, no significant differences in the FoxP3^+^ Treg/CD4^+^T ratio, Tr1 Treg/CD4^+^T ratio, and the Tr1/FoxP3^+^ Treg ratio. The data were shown as dot plots with ± SD of each group. *N* = 10 for MG group and *N* = 11 for NC group.

The therapeutic administration of Tregs after disease induction has shown ameliorating effects (3). It is noted that in MG patients, if enough FoxP3^+^ Tregs are produced to against autoreactive T cells, the disease will be alleviated (2). In MG patients, changes in the function and concentration of FoxP3^+^ Tregs may be one of the important mechanisms of MG pathogenesis (2). Our study suggests that the percentages of FoxP3^+^ Tregs are not significantly different between gMG patients and HCs (Figure 5).

**Figure 6:**
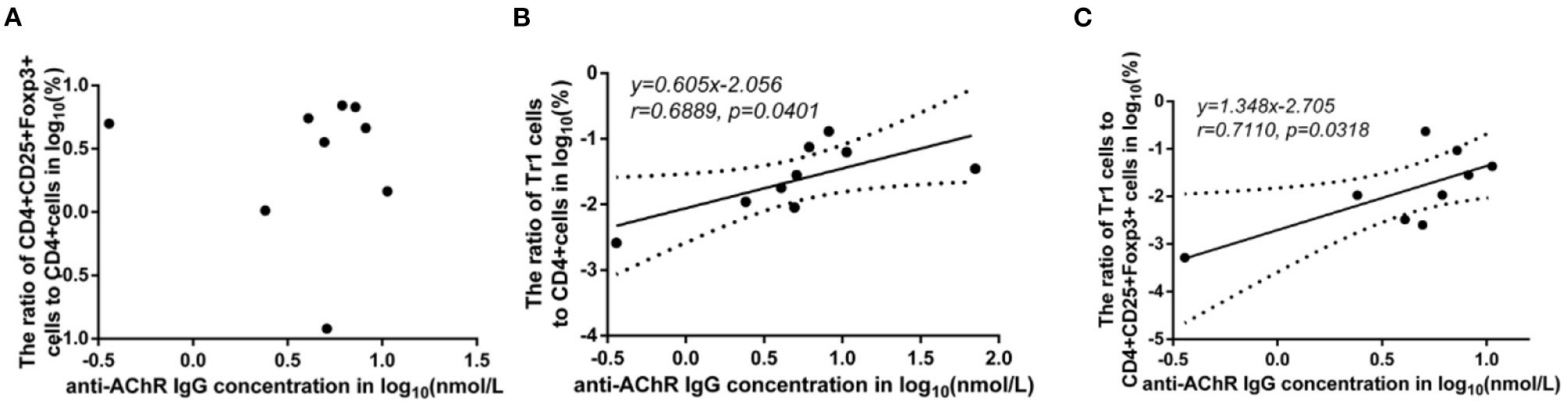
Association between the anti-AChR Ab levels and the ratios of Treg subgroups in MG patients. Shapiro-Wilk's tests were used to assess normal distribution of the data. We identified one outlier using outlier boxplot tool, and this outlier was omitted from graphical presentation of the data, with no effects on the statistical results. Linear regressions (solid black lines) with 95% CI (dotted lines) were conducted to analyze the relationship between the anti-AChR Ab levels and the ratios of Treg subgroups in MG patients, and slopes were compared to 0 in addition to Spearman's correlation test in order to access correlations between variables. **(A)**, no significant correlation between anti-AChR Ab concentration and FoxP3^+^ Treg/CD4^+^T ratio. **(B)**, a positive correlation with statistically significant was found between Tr1/CD4^+^T cell ratio and the level of anti-AChR Ab (*r* = 0.6889 ± 0.4414, *p* = 0.0401). **(C)**, a positive correlation with statistically significant was discovered between Tr1/FoxP3^+^ Tregs cell ratio and the level of anti-AChR Ab (*r* = 0.7110 ± 0.4227, *p* = 0.0318). *N* = 9 for MG patients (one outlier was omitted).

Some studies have observed that the percentages of Tr1 cells among total Treg cells in MG patients were significantly lower than those in healthy controls (3). In our study, although the proportion of Tr1 cells to CD4^+^ T cells in gMG patients tended to be slightly lower than normal controls, it was not significantly different between gMG patients and HCs (*p* = 0.217) (Figure 5C). Therefore, we believe that the difference in the concentrations of different subgroups of Tregs cannot fully reflect the important role of these cell subgroups in the immune regulation of MG. However, it is interesting that as an important factor in the pathogenesis of MG, anti-AChR Ab showed significant positive correlation with Tr1 cells instead of FoxP3^+^ Tregs (Figure 6), suggesting that as immune regulatory cells, Tr1 cells may serve as a powerful supplement to the Treg cell population and play a crucial role in regulating the immune response induced by anti-AChR Ab. However, the mechanism by which Tr1 cells exert their immunomodulatory effects is not yet fully understood. Therefore, the exploration of Tr1 cell-related cytokine levels is necessary.

Changes in the levels of cytokines, especially TGF-β and IL-10, have been proved playing a key role in the self-regulation of many autoimmune diseases (13). Their decline is not only related to the pathogenesis of autoimmune diseases, upregulation of their levels could also promote the immune regulation in autoimmune diseases (14). Previous studies have shown that TGF-β and IL-10 are the main cytokines secreted by Tr1 cells and mediate the immunosuppressive effect of Tr1 cells (18). However, the relationship between Tregs subsets and immunoregulatory cytokines in patients with MG is still controversial. Elevated levels of proinflammatory cytokines, such as IL-6, IL-17, and IFN-γ, secreted by T effectors have been observed in MG patients. Normally, Tregs suppress the production of these proinflammatory cytokines through the contact-dependent and contact-independent suppression of Teff cells known to produce IL-6, IL-17, and IFN-γ (1, 10).

FoxP3^+^ Tregs play an essential role in downregulating pathological T cell responses. But in our study, FoxP3^+^ Tregs are not only not related to the level of anti-AChR Ab (Figure 6A), but also not significantly related to the level of cytokines that have immunomodulatory effects such as IL-10 (Figure 2B). Some studies have shown that the secretion function of FoxP3^+^ Tregs is seriously destroyed in MG patients (2). Another study showed that Tr1 cells play an anti-inflammatory role via their production of IL-10 (15). Tr1 cells produce the immunosuppressive cytokines IL-10 and TGF-β, do not constitutively express FoxP3, and suppress the function of effector immune cells (10–12, 15). However, in our research, we found that as the Tr1 cell to CD4^+^ T cell ratio increased, the level of IL-10 also showed an upward trend (Figure 2C). However, FoxP3^+^ Tregs were not significantly related to changes in the levels of TGF-β or IL-10. This finding suggests that in gMG patients, as an important supplement of FoxP3^+^ Tregs, Tr1 cells play an important role in immune regulation in patients with MG, and in this immune regulation process, IL-10 may act as a powerful supplement to TGF-β. And we hypothesize that there is a feedback regulation under the proinflammatory stimulation. To be specific, as a protective mechanism in MG patients, when the levels of pro-inflammatory pathway markers represented by AchR-Ab and IL-10 increased, the response of Treg cells including Foxp3+ Treg and Tr1 cells were correspondingly elevated, presenting with the increase level of secreted cytokines. And this process may help MG patients restore the immune homeostasis (19). However, evaluation of this hypothesis needs further studies in the future. In this study, we found anti-AChR Ab levels significantly were positively correlate to Tr1 cell concentrations, but not to FoxP3^+^ Treg concentrations in gMG patients, suggesting that Tr1 cells but not FoxP3^+^ Tregs are critical in the process of immune regulation in gMG patients. During this process, IL-10 was downregulated, and positively correlated to Tr1 cells and anti-AChR Ab levels. Our study indicated the importance of Tr1 cells in MG pathogenesis, bringing new hints to elaborate mechanism upon AChR-related autoimmune responses.

## Data Availability Statement

The original contributions presented in the study are included in the article/supplementary material, further inquiries can be directed to the corresponding author/s.

## Author Contributions

HM and SZ participated in writing of the paper. QZ participated in the flow cytometry and data analysis. YG and YN participated in collecting the information of the paper. HL participated in ELISA procedure. HM participated in the clinical data analysis. SC participated in the revising of the paper. All authors contributed to the article and approved the submitted version.

## Conflict of Interest

The authors declare that the research was conducted in the absence of any commercial or financial relationships that could be construed as a potential conflict of interest.

## Publisher's Note

All claims expressed in this article are solely those of the authors and do not necessarily represent those of their affiliated organizations, or those of the publisher, the editors and the reviewers. Any product that may be evaluated in this article, or claim that may be made by its manufacturer, is not guaranteed or endorsed by the publisher.
